# Multi-parameter viscoelastic material model for denture adhesives based on time-temperature superposition and multiple linear regression analysis

**DOI:** 10.1186/s42490-024-00083-z

**Published:** 2024-09-02

**Authors:** Anantha Narayanan Ramakrishnan, Josephine Reymann, Christopher Ludtka, Andreas Kiesow, Stefan Schwan

**Affiliations:** 1 Department of Engineering and Natural Sciences, University of Applied Sciences, Hochschule Merseburg, Merseburg, Germany; 2 Institute for Modelling and Simulation of Biomechanical Systems, Faculty of Civil and Environmental Engineering, University of Stuttgart, Stuttgart, Germany; 3 Department of Biological and Macromolecular Materials, Fraunhofer Institute for Microstructure of Materials and Systems IMWS, Halle (Saale), Germany; 4 Department of Operative Dentistry and Periodontology, Martin-Luther-University Halle-Wittenberg, Halle, Germany; 5 J. Crayton Pruitt Family Department of Biomedical Engineering, University of Florida, Gainesville, USA

**Keywords:** Viscoelastic behavior, Denture adhesives, Temperature, Adhesive swelling, pH, Rheology, Multi-parameter linear regression analysis, Time-temperature superposition, Material modelling

## Abstract

**Background:**

Restorative solutions designed for edentulous patients such as dentures and their accompanying denture adhesives operate in the complex and dynamic environment represented by human oral physiology. Developing material models accounting for the viscoelastic behavior of denture adhesives can facilitate their further optimization within that unique physiological environment. This study aims to statistically quantify the degree of significance of three physiological variables - namely: temperature, adhesive swelling, and pH - on denture adhesive mechanical behavior. Further, based on these statistical significance estimations, a previously-developed viscoelastic material modelling approach for such denture adhesives is further expanded and developed to capture these variables’ effects on mechanical behavior.

**Methods:**

In this study a comparable version of Denture adhesive Corega Comfort was analysed rheologically using the steady state frequency sweep tests. The experimentally derived rheological storage and loss modulus values for the selected physiological variables were statistically analyzed using multi parameter linear regression analysis and the Pearson’s coefficient technique to understand the significance of each individual parameter on the relaxation spectrum of the denture adhesive. Subsequently, the parameters are incorporated into a viscoelastic material model based on Prony series discretization and time-temperature superposition, and the mathematical relationship for the loss modulus is deduced.

**Results:**

The results of this study clearly indicated that the variation in both the storage and loss modulus values can be accurately predicted using the oral cavity physiological parameters of temperature, swelling ratio, and pH with an adjusted *R*^*2*^ value of 0.85. The *R*^*2*^ value from the multi-parameter regression analysis indicated that the predictor variables can estimate the loss and storage modulus with a reasonable accuracy for at least 85% of the rheologically determined continuous relaxation spectrum with a confidence level of 98%. The Pearson’s coefficient for the independent variables indicated that temperature and swelling have a strong influence on the loss modulus, whereas pH had a weak influence. Based on statistical analysis, these mathematical relationships were further developed in this study.

**Conclusions:**

This multi-parameter viscoelastic material model is intended to facilitate future detailed numerical investigations performed with implementation of denture adhesives using the finite element method.

## Background

Denture adhesives are often prescribed by oral healthcare providers, as they can improve the masticatory performance of dentures [1]. These adhesives are primarily composed of bonding agents such as carboxymethylcellulose, which enhances adhesion between the oral mucosa and denture surface under the influence of saliva [2]. Hence, denture adhesives have been recorded to increase retention and stabilization behavior of various types of denture designs, facilitating better overall denture performance [1, 3–5]. Moreover, denture adhesives have also been shown to mitigate denture displacement during the application of biting and chewing forces [6–8]. This tendency for denture adhesives to help hold the denture within its specified functional region of the oral cavity has also been illustrated using numerical studies based on the finite element method (FEM) [9, 10]. FEM-based numerical simulations provide the opportunity for detailed and repetitive assessment of the mechanical behavior of denture adhesives while considering a multitude of other factors that affect the adhesive’s function. Such insights can help optimize denture design or support oral healthcare providers. Numerical studies require that the denture adhesive be implemented using relevant material laws, which allows researchers to better understand the behavior of the denture adhesive material under various bite loads. The results can be used to optimize the composition of denture adhesive’s formulation or to characterize its impact on the oral health of the denture wearers.

Denture adhesives are expected to function under the highly dynamic and variable physiological conditions within the oral cavity. Several previous reports have indicated the dependence of denture adhesive performance on several of the physiological variables prevalent in the oral cavity like temperature, pH, and saliva [11–16]. Nevertheless, both the degree of impact and the relative significance of these individual variables have not been investigated in great depth. Doing so could potentially provide further information towards developing a dedicated mathematical model or formulae for such materials. The role of several independent variables in estimating an output variable – like the storage modulus or the loss modulus, which can define material behavior – can be studied using statistical techniques such as multiple linear regression analysis or multiple non-linear regression analysis [17, 18]. Specifically, the magnitude of energy stored in a material is described by its storage modulus (G′), and the energy lost due to dissipation is described by its loss modulus (G″). Further, the Pearson’s correlation coefficient can be used to measure the strength of the linear relationship between these two sets of variables [19, 20]. The Pearson’s coefficient can have values from − 1 to 1, with − 1 implying a completely negative correlation and 1 implying a completely positive correlation [21].

Many polymeric materials such as pastes and adhesives have been shown to exhibit time-dependent and rate-dependent material responses [22]. Denture adhesives in particular have exhibited such viscoelastic mechanical behavior, especially at higher temperatures [23, 24]. Numerical implementation of the continuous relaxation spectrum obtained through rheological measurements requires its approximation using a discrete number of measurement points, which is possible using Prony series discretization [25]. Ramakrishnan et al. (2023) used such rheological measurements and presented a viscoelastic material model based on the Prony series discretization approach for denture adhesives [26]. Therein, the experimental results for the adhesive were utilized to model a viscoelastic material based on the Prony series approximation technique with a specified number of Prony series terms. Equation 1 represents the result from that study for the shear relaxation modulus, *G*, in terms of the relaxation time, $\tau$. Further, $\theta$ represents the temperature in the oral cavity and ${G}_{0}$ represents the intercept [26].

1 $$\varvec{G} \left(\varvec{t}, \varvec{\theta }\right)={\varvec{G} \left(\varvec{t}\right)=\varvec{G}}_{0 }\varvec{e}\varvec{x}\varvec{p} \left(-\frac{\varvec{t}}{{\varvec{\tau }}_{\varvec{\theta }}} \right)$$

The present work was further aimed to statistically analyze and interpret the impact of the oral cavity’s physiological parameters - namely: temperature, pH, and swelling due to saliva - on the mechanical behavior of denture adhesives. The multiple linear regression analysis was performed based on the null hypothesis that the predictor variables temperature, swelling ratio and pH do not influence the response variables, i.e., the storage modulus and the loss modulus of the denture adhesive. Additionally, based on the significance of these independent variables, they were incorporated into a multi-parameter viscoelastic material model based on Prony series discretization and time-temperature superposition in order to perform numerical investigations using the finite element method.

## Methods

In order to develop the viscoelastic material modelling approach for denture adhesives as well as incorporate further physiological parameters into this model, the current study starts with the basic model previously illustrated in the work of Ramakrishnan et al. 2023 [26]. That study focused on mapping three prominent physiological variables of the oral cavity: the temperature of the oral cavity, the pH of the medium, and the swelling of the denture adhesive due to saliva. Rheological steady state shear tests provided the relaxation spectra for the specific denture adhesive which was comparable in composition to Corega Comfort manufactured by GSK Oral Health was tested for different values of temperature, pH, and swelling ratio. The denture adhesive was mainly composed of Calcium/Sodium PVM/MA Copolymer, Petrolatum, Cellulose Gum as active ingredient and Paraffinum Liquidum as inactive ingredient. The methodology for sample preparation and rheological test performance were based on the work of Gill et al. 2017 [27]. The denture adhesive was quantified using a rotational rheometer with parallel plate configuration at a shear rate of 0.3% and over a frequency range of 0.01 Hz to 10 Hz with 10 measurement points for each decade of the interval. The denture adhesive was studied at temperatures from 17 °C to 52 °C in steps of 5 °C. Further, the rheological tests were also conducted for the adhesive maintained at three different pH values (2, 7 and 10) and when the adhesive had attained specific levels of swelling till saturation (in steps of 20%). As a result, Ramakrishnan et al. 2023 [26] reported the experimentally-determined storage and loss modulus values for the specific denture adhesive formulation that were subsequently used in the present study. Based on these rheological results for the storage modulus and the loss modulus at various temperatures, pH and levels of swelling a basic model for numerical simulations was proposed in the work of Ramakrishnan et al. 2023 [26] which is demonstrated in Eq. 1. The influence of the noted physiological variables on the denture adhesive were further captured into a basic viscoelastic material modelling approach based on Prony series discretization.

### Statistical analysis

Before developing the viscoelastic material model further and incorporating the impact of the individual physiological variables into it, the significance of each individual variable on the relaxation spectrum was statistically determined. For this purpose, a multiple linear regression analysis was performed considering the entire domain of pH, swelling, and temperature on both the storage and loss moduli. The analysis was conducted with a confidence level of 98%, and the probability of seeing a response described by the p − value of the variables was compared. For consideration of the null hypothesis that the predictor variables of temperature, swelling, and pH do not influence the response variables (i.e. the storage and loss moduli), a multiple linear regression test was performed. Following the regression study, the covariance of the parameters was explored using Pearson’s correlation coefficient, also called Pearson’s *R*. The Pearson’s coefficient of each independent oral cavity physiological variable with respect to the dependent variables of storage and loss moduli was compared and analyzed to characterize the specific denture adhesive formulation investigated. Based on the regression tests and the Pearson’s coefficient values for the individual parameters, their relative significance and impact on the material behavior was assessed and discussed.

### Time-temperature superposition

The observed relaxation spectra indicated a horizontal shift with variation of the temperature during rheological testing. The horizontal shift factors were evaluated using the Arrhenius shift factor, as previously illustrated [26]. Based on the foundation towards a viscoelastic material model introduced in the work of Ramakrishnan et al. 2023 [26], and using the time-temperature superposition approach also discussed in that work, the parameter ${a}_{\theta }$, which represents the horizontal shift factor, was introduced into the mathematical expression to obtain the relationship of relaxation modulus in terms of both the relaxation time, $\tau$, and the oral cavity temperature, $\theta$. The variables of pH and swelling ratio were also incorporated into the viscoelastic material model based on their relaxation amplitudes and the relation times of the corresponding elements of the Prony series discretization with *n* = 3 terms. The choice of the number of terms of the Prony series discretization was based on the accuracy of the fit curve drawn using OriginPro 2019 (OriginLab Corporation, MA United States) based on the relaxation spectrum of the denture adhesive.

## Results

The results for the multiple linear regression analysis are presented in Table 1 for the experimentally-determined storage modulus values with regards to the three independent variables of temperature, pH, and swelling ratio of the denture adhesive that were considered in this study. For both temperature and swelling ratio the p − value was observed to be < 0.0001 for prediction of the storage modulus values. The observed p-values for all three variables were observed to be less than 0.02, which was the threshold for significance based on the confidence level of the test being set at 98%. Similarly, for the case of the loss modulus, the multiple linear regression analysis results are compiled in Table 2. Again, both swelling and temperature exhibited a probability value (*p* < 0.0001) indicating that the null hypothesis could be rejected here as well.

**Table 1:** The results from multiple linear regression analysis of the storage modulus values for the system of variables in this study, evaluated at a confidence level of 98%

	Parameter	t value	Pr > |t|	Prediction Confidence	Significance Level
1	Oral Temperature	-16.604	< 0.0001	> 99.9999%	Highly Significant
2	pH	-0.853	< 0.001	> 99.999%	Significant
3	Swelling ratio of Adhesive	-5.135	< 0.0001	> 99.9999%	Highly Significant
4	Intercept Term	47.561	< 0.0001	> 99.9999%	Highly Significant

Confidence level 98% and significance level of 0.02

**Table 2:** The results from multiple linear regression analysis of the loss modulus values for the system of variables in this study, evaluated at a confidence level of 98%

	Parameter	t value	Pr > |t|	Prediction Confidence	Significance Level
1	Oral Temperature	-7.721	< 0.0001	> 99.9999%	Highly significant
2	pH	0.965	< 0.001	> 99.999%	Significant
3	Swelling ratio of Adhesive	4.285	< 0.0001	> 99.9999%	Highly significant
4	Intercept Term	30.033	< 0.0001	> 99.9999%	Highly significant

Confidence level 98% and significance level of 0.02

**Fig. 1:**
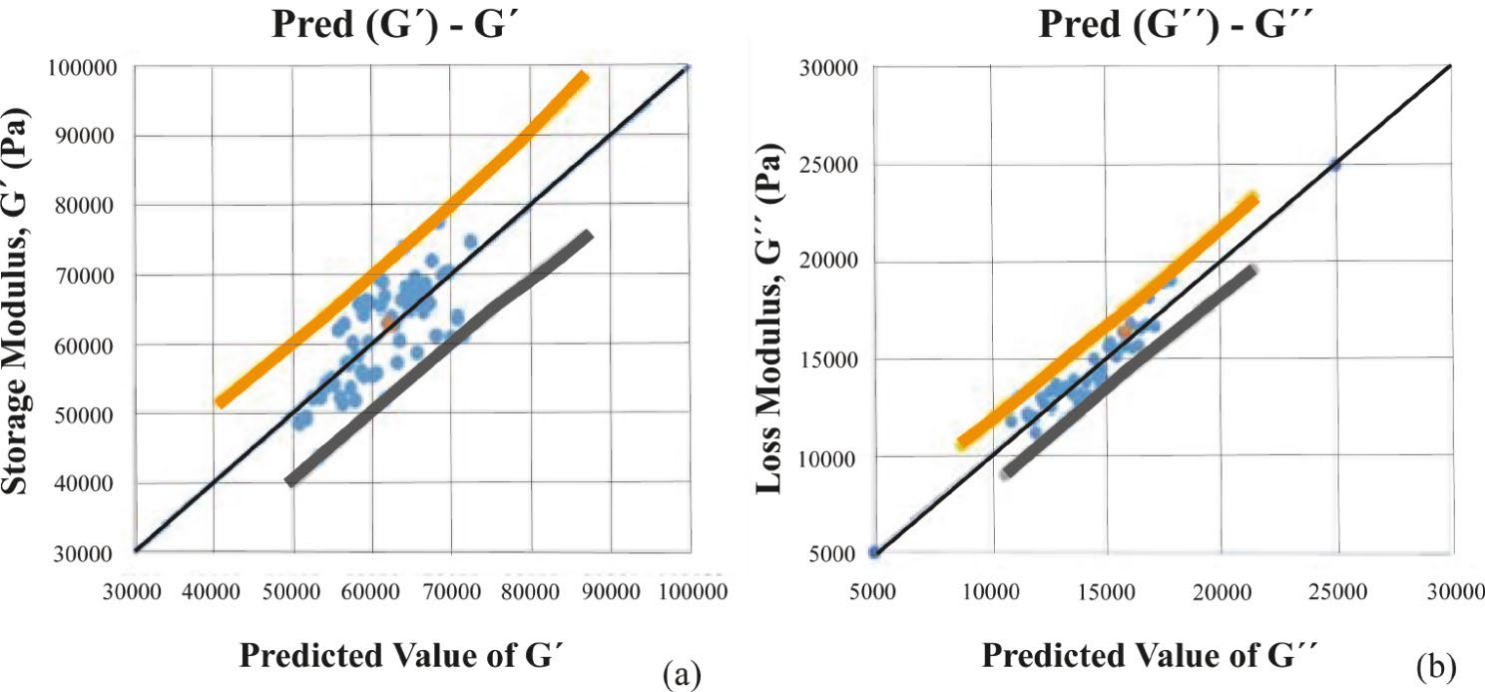
Graphical illustration of the multiple linear regression analyses performed using the three independent variables of temperature, pH, and swelling ratio in order to predict the value of the (**a**) storage modulus and (**b**) loss modulus of the tested denture

Figure 1(a) gives a graphical view of the multiple linear regression analysis performed for the storage modulus of the denture adhesive specimen tested in this study, while Fig. 1(b) does the same for the loss modulus values.

The covariance of the oral cavity’s physiological parameters relative to one another using the Pearson’s *R* is presented in Table 3 for both the storage modulus and loss modulus values. The temperature showed a higher Pearson’s *R* value for both the response variables (i.e. storage and loss modulus). The swelling ratio also showed a strong correlation to both the storage and loss modulus values. The pH on the other hand indicated a weaker correlation to the two dependent variables when compared with the temperature and swelling ratio.

**Table 3:** The results of the Pearson’s correlation matrix for the independent physiological variables and the corresponding dependent modulus variables

	Variables in the study	Swelling Ratio	Temperature	pH	Storage Modulus, G´	Loss Modulus, G´´
1	Swelling Ratio	1	0	0	-0.269	0.361
2	Temperature	0	1	0	-0.869	-0.65
3	pH	0	0	1	-0.045	0.078
4	Storage Modulus, G´	-0.269	-0.869	-0.045	1	0
5	Loss Modulus, G´´	0.361	-0.65	0.078	0	1

**Fig. 2:**
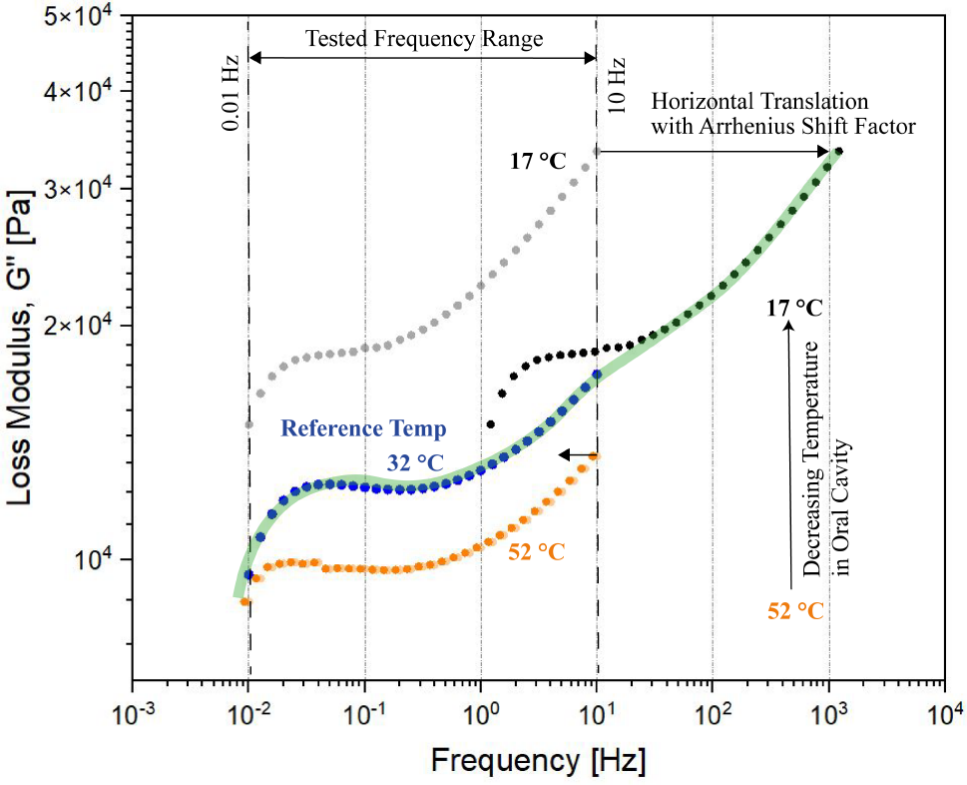
The time-temperature superposition performed on the relaxation spectra based on the calculated Arrhenius shift factors for the individual relaxation spectra at the specific temperatures that were evaluated

After the statistical interpretation of the rheological results from the frequency sweep test the individual plots at varying temperatures were subjected to time-temperature superposition and the corresponding calculated Arrhenius shift factors are illustrated in the work of Ramakrishnan et al. 2023 [27]. Figure 2 illustrates the application of the calculated horizontal shift factors to the individual relaxation spectrum, using the relaxation profile observed at 32 °C as the reference curve for performing the horizontal shift.

## Discussion

The behavior of polymeric materials like denture adhesives can be satisfactorily estimated in most cases using the relaxation spectrum, and this data can be used as a foundation for any other standard mechanical tests [28]. In this study, the rheological measurements for a denture adhesive comparable to Corega Comfort was analyzed statistically using the multiple linear regression analysis. Further, the influence of physiological variables in the oral cavity on the denture adhesive was modeled mathematically resulting in the proposal of a viscoelastic multiparameter material model. Based on the rheological experiments, the storage modulus of the specific denture adhesive cream investigated was observed to be higher than the loss modulus across the considered range of test variables used. Denture adhesives, like any material, exhibit elastic properties when G′ < G″ and viscous properties when G′ > G″ [29]. As the G″ curve crosses over the G′ curve, a point it reached where the material starts to flow. Based on the rheological results and the multiple linear regression analysis described in Tables 1 and 2 for the three independent variables of temperature, pH, and swelling ratio, it was inferred that the three variables could be used to predict both the storage and loss modulus values with a fair degree of accuracy. The loss modulus plot had a regression parameter *R*^*2*^ value of 0.85, which considering the large variability and complexity of the test specimen, indicates a good level of prediction of the loss modulus. This implies that approximately 85% of the variation in the loss modulus profile can be meaningfully estimated using the predictor variables of temperature, pH, and swelling ratio. Additionally, the highest residuals were observed for only a few cases, the exclusion of which increased the *R*^*2*^ value to approximately 0.95, which was strongly predictive [30]. The p − value for temperature, swelling ratio and the pH were observed to be < 0.0001 for both the storage modulus and loss modulus values. Therefore, the null hypothesis was successfully rejected as the observed p − values were much lower than the significance level of 0.02 based on the 98% confidence test for all the variables in this study, i.e., temperature, swelling and pH.

**Fig. 3:**
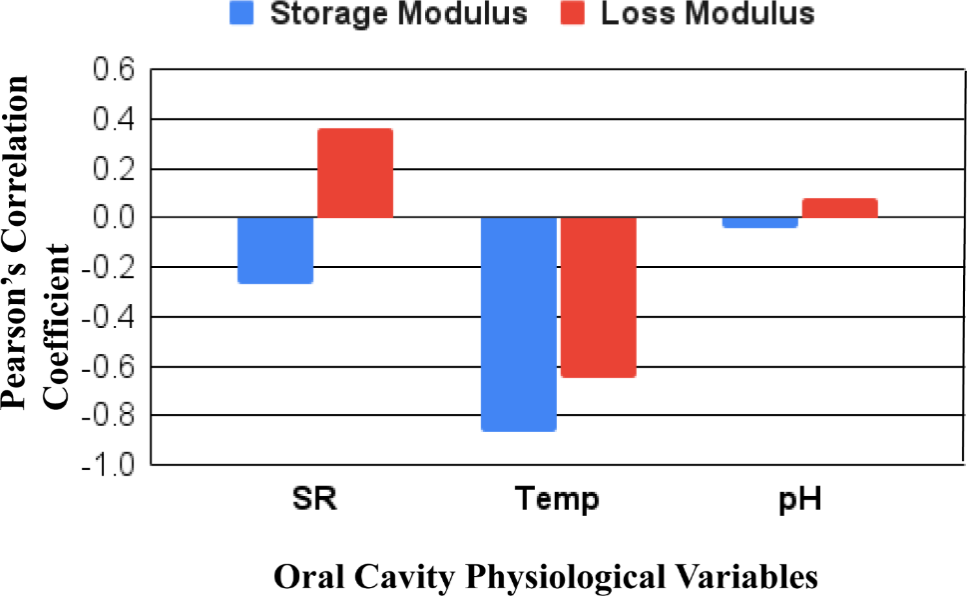
Pearson’s correlation matrix for the dependent variables of storage modulus and loss modulus for the three given independent physiological variables of the oral cavity. SR = swelling ratio. Temp = temperature

The Pearson’s correlation matrix is illustrated graphically in Fig. 3 based on the results from Table 3 for both the storage and loss modulus values. Based on the Pearson’s coefficient values it can be assumed that the temperature and swelling ratio had a stronger linear correlation to both the dependent variables (i.e. the storage and loss modulus) for the specific denture adhesive formulation evaluated. The slightly higher correlation of the swelling ratio with the loss modulus compared to its impact on the storage modulus is potentially meaningful, as the loss modulus better describes the adhesive material. As observed both in Tables 3 and Fig. 3, the swelling ratio had a direct positive correlation with respect to the storage modulus, G’, and an inverse negative effect on the loss modulus, G”. For pH, the correlation also demonstrated a similar inversion, although the degree of correlation was observed to be much lower compared to both temperature and swelling ratio. However, it is important to note that the experimental data measuring the influence of pH was limited to three discrete values: pH 2 for the acidic range, pH 7 for the neutral range, and pH 10 for the alkaline range of the measurement spectrum. These three data points were statistically limited, and hence, the impact of pH on the shear moduli must be further experimentally investigated to more confidently assimilate it into the numerical material model.

The shift factor, ${a}_{\theta }$, is a function of temperature, $\theta$, and takes into account the shift in the continuous relaxation spectrum with each change in temperature. Based on the above deductions, the relaxation modulus, $G$, could thus be evaluated using the mathematical expression indicated in Eq. 2. Here, ${\prime }{G}_{0}{\prime }$ is the instantaneous component of the relaxation spectrum and ${g}_{i}$ are the amplitudes of the $i$^th^ element of the Prony series approximation [26].

2 $$\mathbf{G} \left(\mathbf{t}, \varvec{\uptheta }\right)={\mathbf{G}}_{0}+ \sum _{\mathbf{i}=1}^{\mathbf{n}}{\mathbf{g}}_{\mathbf{i}} \mathbf{e}\mathbf{x}\mathbf{p} \left(-\frac{{\mathbf{a}}_{\varvec{\uptheta }}\left(\varvec{\uptheta }\right)\mathbf{t}}{{\varvec{\uptau }}_{\mathbf{i}}} \right)$$

Both the impact of swelling ratio of the denture adhesive due to its interaction with saliva as well as the pH of the medium were taken into account in this study in an approach similar to that of Ramakrishnan et al. 2023 [26]. The relaxation times, ${\varvec{\uptau }}_{\mathbf{i}}$, for the $i$^th^ element of the Prony series approximation and the corresponding amplitudes, ${g}_{i}$, for a given time, $t$, and temperature,$\theta$, varies with both swelling ratio and the pH, as illustrated in the results. Taking this variation into account in the overall expression leads to the shear relaxation modulus for the denture adhesive taking the form of Eq. 3. In this expression, we define ${\left({\tau }^{ SR, pH}\right)}_{i}$ as the approximated Maxwell model’s relaxation time for its $i$^th^ branch, which is used to mathematically represent the denture adhesive formulation that has attained a swelling ratio, $SR$, and is at a particular pH value, $pH$. Similarly, ${\left({g}^{ SR, pH}\right)}_{i}$ is defined as the amplitude of the $i$^th^ branch of the corresponding Maxwell model approximation of the denture adhesive at the same swelling ratio, $SR$, and operating at the specific pH value, $pH$.

3 $$\varvec{G} \left(\varvec{t}, \varvec{\theta }, \varvec{S}\varvec{R}, \varvec{p}\varvec{H}\right)={\varvec{G}}_{0}+ \sum _{\varvec{i}=1}^{\varvec{n}}\left[{ \left({\varvec{g}}^{ \varvec{S}\varvec{R}, \varvec{p}\varvec{H}}\right)}_{\varvec{i}} \mathbf{e}\mathbf{x}\mathbf{p}\left(-\frac{{\varvec{a}}_{\varvec{\theta }}\left(\varvec{\theta }\right)\varvec{t}}{{\left({\varvec{\tau }}^{ \varvec{S}\varvec{R}, \varvec{p}\varvec{H}}\right)}_{\varvec{i}}} \right)\right]$$

As can be interpreted from the statistical results, the influence of both temperature and swelling ratio were classified as very significant. The pH value however only showed a weak correlation to both the storage and loss modulus values based on the Pearson’s coefficient values. Hence, the equation was simplified by ignoring the pH parameter, which results in Eq. 4. Equation 3 and Eq. 4 are hypothesized to capture the effect of all three physiological variables (i.e. temperature, pH, and swelling ratio) satisfactorily into the viscoelastic material model of the denture adhesive, and therefore can be used for performing numerical simulations with FEM using standard commercially available software packages.

4 $$\varvec{G} \left(\varvec{t}, \varvec{\theta }, \varvec{S}\varvec{R}\right)={\varvec{G}}_{0}+ \sum _{\varvec{i}=1}^{\varvec{n}}\left[\begin{array}{c}{\left({\varvec{g}}^{ \varvec{S}\varvec{R}}\right)}_{\varvec{i}} ex\mathbf{p}\left(-\frac{{\varvec{a}}_{\varvec{\theta }}\left(\varvec{\theta }\right)\varvec{t}}{{\left({\varvec{\tau } }^{\varvec{S}\varvec{R}}\right)}_{\varvec{i}}} \right)\\ \end{array}\right]$$

The study limits the model to the influence of temperature, pH, and swelling of the denture adhesive and neglects the role of other parameters in the oral cavity. The consideration of these specific variables was based on the crucial role played by saliva and the inherent viscosity of the adhesive. We argue that the viscosity is crucial in understanding the resultant retention behavior and hence, the temperature and swelling under the influence of saliva were considered. Further in order to include the large variations in pH with the different food samples consumed, the pH was also included in this study. The material model based on Eqs. 3 and 4 can facilitate in performing numerical simulations using the FEM which can potentially aid the dental practitioners in supporting the patients. Although the denture adhesives are temporary materials, they can contribute significantly to the contact stresses developed on the soft tissue and the associated dental structures as demonstrated in previous studies [9, 31]. These studies approximate the denture adhesives as simple materials and the proposed model based on Eqs. 3 and 4 can be argued to substantially enhance the details regarding the stress state in the denture - soft tissue interfaces. Hence, this study proposes a multi parameter modeling approach for the denture adhesives which can then facilitate towards the development of holistic FEM based models that can be potentially used for patient specific care in the future.

## Conclusion

This study advances the development of an exemplary multi-parameter viscoelastic material model for denture adhesive formulations. Based on Eq. 3 or Eq. 4 the mechanical behavior of the denture adhesive can potentially be characterized under the influences of temperature, swelling ratio, and pH using four unknowns, namely: the number of Prony series terms, *n*; the relaxation time, ${\left({\tau }^{ SR, pH}\right)}_{i}$; the amplitude of the corresponding branch,${ \left({g}^{ SR, pH}\right)}_{i}$; and the Arrhenius shift factors of the relaxation spectra. These parameters are in turn dependent on the temperature, pH, and swelling ratio of the denture adhesive. This model can be implemented with standard, commercially-available FEM packages with slight modifications that are software specific. For instance, in the software package ANSYS the above viscoelastic material model with time-temperature superposition can be implemented using the Tool ‘Narayanaswamy shift function’, which is a simplification of the Arrhenius shift function [32] and the Prony series method. This model has the potential to be developed further to include many more parameters that were beyond the scope of the present study in order to optimize denture adhesives as a material to best facilitate denture wearers.

## Data Availability

The datasets used and/or analysed during the current study are available from the corresponding author on reasonable request.
